# Mandibular bone regeneration after marginal mandibulectomy for advanced MRONJ following denosumab discontinuation: a case report

**DOI:** 10.3389/froh.2026.1848520

**Published:** 2026-06-12

**Authors:** Wataru Kotani, Chonji Fukumoto, Toshiki Hyodo, Yuske Komiyama, Tomonori Hasegawa, Takahiro Wakui, Hitoshi Kawamata

**Affiliations:** 1 Department of Oral and Maxillofacial Surgery, Dokkyo Medical University School of Medicine, Mibu, Tochigi, Japan; 2 Ichigaya Concier Dental Clinic, Chiyoda-ku, Tokyo, Japan; 3 Section of Dentistry, Oral and Maxillofacial Surgery, Sano Kosei General Hospital, Sano, Tochigi, Japan

**Keywords:** bone metastasis, bone regeneration, bone-modifying agents (BMA), denosumab, medication-related osteonecrosis of the jaw (MRONJ)

## Abstract

**Background:**

Medication-related osteonecrosis of the jaw (MRONJ) is a serious complication associated with bone-modifying agents, including denosumab. The clinical course after denosumab discontinuation remains insufficiently understood, and optimal surgical strategies for advanced cases are still under debate.

**Case presentation:**

We report a 71-year-old man with castration-resistant prostate cancer and multiple bone metastases who developed MRONJ during denosumab therapy for more than 3 years. After diagnosis at our institution, denosumab was discontinued based on the decision of the treating oncologist. Rapid progression of bone resorption was observed within several months, in which discontinuation of denosumab may have been a contributing factor. Although segmental mandibulectomy was considered, the patient declined radical surgery. Marginal mandibulectomy was therefore performed via an intraoral approach, with preservation of the lingual bone plate and periosteum. During long-term follow-up, marked bone regeneration became evident by 2 years postoperatively and was sustained at 6 years, with restoration of mandibular continuity. No recurrence of MRONJ or new skeletal-related events was observed during ongoing oncological follow-up.

**Conclusion:**

Discontinuation of denosumab may have contributed to rapid osteolytic progression in MRONJ. Preservation of biological structures during marginal mandibulectomy, as part of multidisciplinary management, may be associated with favorable bone regeneration. These findings highlight the complexity of MRONJ management and the importance of individualized, multidisciplinary approaches.

## Introduction

Medication-related osteonecrosis of the jaw (MRONJ) is a well-recognized complication associated with bone-modifying agents (BMAs), including bisphosphonates and denosumab (1–3). Patients receive these agents at low doses for the treatment of osteoporosis, including primary and glucocorticoid-induced osteoporosis, and at higher doses for metastatic bone disease in several malignant tumors. The risk of MRONJ differs substantially depending on the indication and dosage of the drugs. In patients receiving low-dose BMAs for osteoporosis, the incidence of MRONJ is generally low, typically ranging from 0.01% to 0.1% (1, 2). In contrast, among cancer patients receiving high-dose BMAs, the reported incidence of MRONJ has been approximately 1%–2% in some studies (1, 2). However, other reports have suggested that the risk may be substantially higher with prolonged exposure; for example, cumulative incidence has been reported to reach 4.7% at 2 years, 18.1% at 5 years, and 32.1% at 8 years in long-term observational studies (4). MRONJ can significantly impair quality of life and complicate the treatment of underlying malignancies (1, 5).

Treating patients with MRONJ remains challenging, particularly in patients with advanced disease requiring surgical treatment (5). In addition, clinical decision-making regarding BMAs for both oral surgeons and medical oncologists, especially the timing of discontinuation and the optimal duration of therapy in patients with stabilized metastatic disease, remains controversial (6). Furthermore, the clinical course of MRONJ after denosumab discontinuation and its response to surgical treatment remains poorly understood. Notably, accelerated osteolytic changes following cessation of denosumab have been suggested, possibly reflecting alterations in bone metabolism; however, clinical evidence remains limited (7).

In this report, we describe the clinical course of a patient with MRONJ in which marked osteolytic progression became evident following denosumab discontinuation. Despite this unfavorable clinical course, substantial bone regeneration was observed during long-term follow-up after marginal mandibulectomy. The clinical course of this patient highlights important considerations regarding the pathophysiological changes associated with MRONJ after denosumab discontinuation, as well as surgical treatment strategies and the optimal duration of denosumab therapy in cancer patients.

## Case presentation

A 71-year-old man was referred to our hospital in March 2019 for evaluation of mandibular gingival swelling. His medical history included castration-resistant prostate cancer with multiple bone metastases involving the skull, spine, sacrum, ribs, pelvis, and long bones of the limbs. He initially presented in 2015 with lower limb numbness and gait disturbance, and was subsequently diagnosed with prostate cancer with multiple bone metastases, with a prostate-specific antigen level exceeding 10,000 ng/mL. He immediately underwent androgen deprivation therapy with a gonadotropin-releasing hormone antagonist, followed by posterior decompression surgery for metastatic tumors of the thoracic spine (T2–T4) performed by the orthopedic surgery department. After transfer for rehabilitation, systemic therapy with enzalutamide and leuprorelin was initiated at another hospital, at which time his prostate-specific antigen level had decreased to approximately 10 ng/mL. He had also received denosumab every three months, with the last dose administered in March 2019. He was taking medication for hypertension and had no history of food or drug allergies. No other medications affecting bone metabolism were used, and no dental evaluation had been performed before the initiation of denosumab therapy. In addition, no regular preventive dental care had been provided.

The patient first noticed mandibular gingival swelling in April 2018 and was treated at a local dental clinic. From approximately July 2018, he developed numbness of the right lower lip and mental region, along with swelling of the left submandibular area. As these symptoms persisted despite repeated local treatments and medication, he was referred to our hospital for further evaluation. At the time of referral, the treating dentist did not appear to have recognized the patient's history of denosumab use or the possibility of MRONJ, while the treating urologist did not appear to have recognized the development of MRONJ.

At the first visit, the patient had persistent lower limb numbness and motor impairment following surgery for spinal metastases in 2015 and was unable to ambulate independently, requiring wheelchair assistance for mobility and assistance with activities of daily living. He was able to consume only soft foods and had experienced an unintentional weight loss of approximately 5 kg since mid-2018. Extraoral examination revealed diffuse swelling with mild erythema and tenderness in the right submandibular region, without fluctuation or fistula formation. Hypoesthesia of the right lower lip and mental region was noted, and maximum mouth opening distance was 30 mm. Intraoral examination showed that all remaining mandibular teeth were mobile and tender to percussion. Multiple fistulas with purulent discharge were observed in the mandibular gingiva, and bone exposure was confirmed by probing.

Panoramic radiograph at the first visit (Figure 1A) demonstrated diffuse sclerosis and irregular trabecular patterns in the posterior mandible bilaterally, without clear evidence of sequestrum. Computed tomography (Figure 1B) showed periosteal new bone formation and diffuse sclerosis of the cancellous bone, along with localized bone resorption around tooth roots. No definite sequestrum-like bone was identified, and mandibular continuity was preserved. His mandibular condition was diagnosed as bilateral medication-related osteonecrosis of the jaw (MRONJ), stage 2, based on the Japanese clinical practice guidelines developed by a joint committee of six academic societies in Japan (8). Although the diagnosis was made according to these Japanese criteria, they are largely consistent with those of the American Association of Oral and Maxillofacial Surgeons (AAOMS) (1), except for the exclusion of stage 0, thereby enabling international comparability. At the first visit, periosteal reaction and hypoesthesia of the lower lip and mental region suggested possible involvement beyond the alveolar bone. However, no definite pathological fracture, extraoral fistula, or extensive destruction involving the basal bone was observed. Therefore, the lesion was clinically classified as stage 2 at that time. Nevertheless, retrospective interpretation of these findings may suggest that more advanced basal bone involvement had already been present.

**Figure 1:**
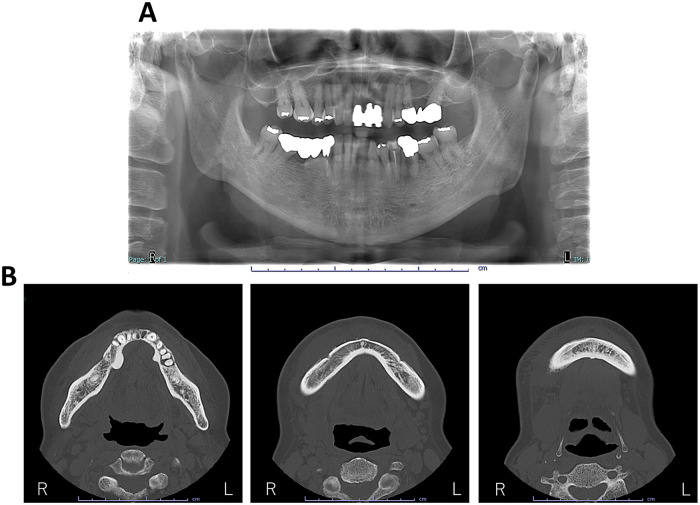
Findings at the first visit, obtained during ongoing denosumab therapy (prior to discontinuation). **(A)** Panoramic radiograph showing diffuse sclerosis and irregular trabecular patterns in the posterior mandible bilaterally, without clear sequestrum-like view. **(B)** Computed tomography showing periosteal new bone formation along the buccal cortex and diffuse sclerosis of the cancellous bone. No definite sequestrum is identified, and mandibular continuity is preserved.

The patient was initially treated with oral amoxicillin (750 mg/day) to control infection-related symptoms, which led to improvement in pain and mouth opening. The antibiotic therapy was continued for approximately one month. Thereafter, preparations for surgery were initiated; however, due to the patient's limited activities of daily living, frequent outpatient visits were difficult. As a result, follow-up visits were conducted approximately once per month until surgery. During the intervals between visits, the patient was instructed to perform regular oral rinsing with a 0.004% benzethonium chloride solution. At each visit, professional oral care was performed, including mechanical cleaning and intraoral cleansing using a 0.025% benzalkonium chloride solution. Surgical treatment under general anesthesia, including extraction of all remaining mandibular teeth and marginal mandibulectomy, was planned. Surgery was scheduled for November 2019, approximately eight months after the first visit. In the meantime, denosumab had been discontinued 6 months prior to surgery based on the decision of the patient's medical oncologist, following communication regarding the development of MRONJ and the current status of the malignant disease.

Several weeks prior to surgery, a cutaneous fistula with purulent discharge had developed in the right submandibular region (Figure 2A), and multiple mandibular teeth had spontaneously exfoliated. Extensive bone exposure and suppuration were present intraorally. Panoramic radiograph (Figure 2B) revealed widespread radiolucent area in the mandible extending from the anterior mandible to the right mandibular body, reaching the inferior border. Computed tomography (Figure 2C) demonstrated extensive osteolysis and separation of sequestrum-like bone in the mandibular body, with findings suggestive of a pathological fracture and disruption of mandibular continuity. The disease was therefore considered to have progressed to stage 3 of MRONJ.

**Figure 2:**
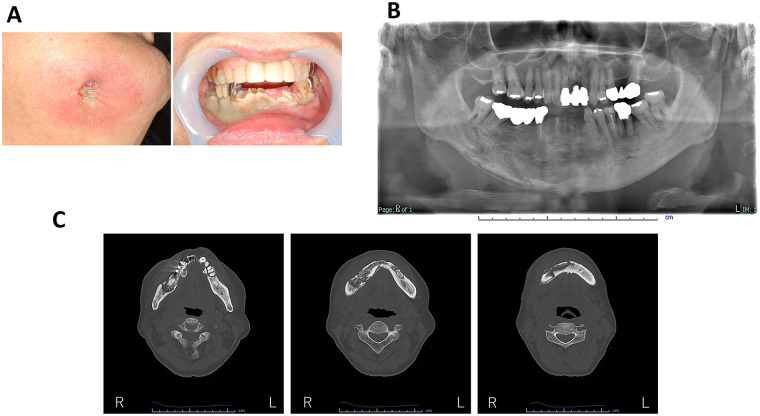
Preoperative findings showing progression of bone resorption. Findings obtained 9 months after the first visit and 6 months after the final dose of denosumab. **(A)** Extraoral and intraoral views showing a cutaneous fistula with purulent discharge in the right submandibular region and extensive intraoral bone exposure. **(B)** Panoramic radiograph showing sequestrum extending from the anterior mandible to the right mandibular body, reaching the inferior border. **(C)** Computed tomography showing widespread osteolysis and sequestrum-like view, with findings suggestive of pathological fracture and disruption of mandibular continuity.

The most appropriate treatment was considered to be segmental mandibulectomy; however, the patient declined radical surgery due to concerns regarding functional impairment and facial deformity. After thorough explanation of the potential risks, including persistent infection and the possible need for secondary surgery, marginal mandibulectomy was selected. Surgery was performed under general anesthesia via an intraoral approach. All remaining mandibular teeth were extracted, and mucoperiosteal flaps were elevated bilaterally. Clinically suspected necrotic bone was carefully removed and curetted, with intentional preservation of not only the periosteum but also the attached cortical bone as much as possible, even when its continuity was disrupted, as part of a conservative surgical approach. On the right side, only the lingual cortex and part of the inferior margin were preserved. The inferior alveolar nerve on the right side was already necrotic, whereas the nerve on the left side was preserved. Residual cortical bone in the right molar region was cracked. After smoothing of sharp bone edges and irrigation, the cutaneous fistula and tract were excised. Additional releasing incisions were required to achieve tension-free closure due to mucosal scarring. A suction drain was placed via the left submandibular region, and complete closure by mucoperiosteal flap was achieved. The operative time was 2.5 h, and blood loss was 300 mL. Histopathological examination (hematoxylin and eosin staining) revealed necrotic bone characterized by empty lacunae and absence of viable osteocytes, with scattered bacterial colonies and inflammatory debris (Figure 3A). A panoramic radiograph obtained immediately after surgery (Figure 3B) showed extensive bone removal from the mental region to the right mandibular body, with only a faint outline of the remaining cortical bone and compromised continuity of the inferior border.

**Figure 3:**
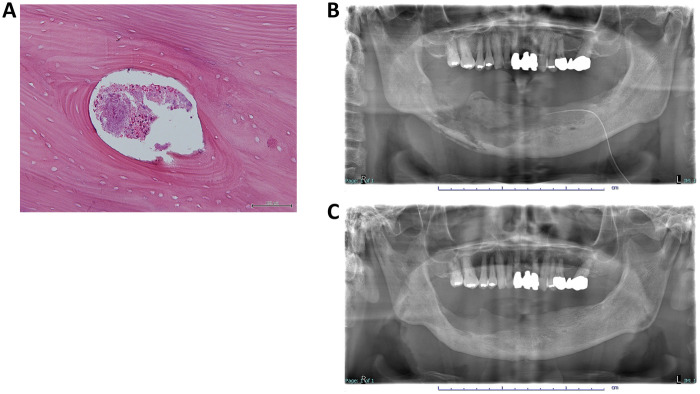
Findings immediately after surgery and at 2 years postoperatively. **(A)**　Histopathological findings (hematoxylin and eosin staining, ×200) showing necrotic bone with empty lacunae and absence of viable osteocytes, accompanied by scattered bacterial colonies and inflammatory debris. **(B)** Panoramic radiograph obtained immediately after surgery showing extensive bone removal from the mental region to the right mandibular body. Only a faint outline of the remaining cortical bone is visible, and continuity of the inferior border appears compromised. **(C)** Panoramic radiograph obtained 2 years postoperatively showing marked bone regeneration of the mandibular body, with restoration of continuity and resolution of the pathological fracture.

Postoperatively, the drain was removed on day 5. Intravenous flomoxef was administered for one week postoperatively, following one week of preoperative administration. Hyperbaric oxygen therapy was initiated two weeks after surgery and was performed for a total of 20 sessions. The patient was discharged approximately one month after surgery. After discharge, oral management was continued in a manner similar to the preoperative period, including regular oral rinsing with a 0.004% benzethonium chloride solution and professional oral care with mechanical cleaning and intraoral cleansing using a 0.025% benzalkonium chloride solution at follow-up visits.

During follow-up, minor wound dehiscence and bone exposure were observed at six weeks postoperatively. Occasionally, small bone fragments were observed at the surgical site, and the fragments were removed and the patient received short-term antibiotics. At one year postoperatively, small areas of mucosal depression with internal bone exposure persisted without signs of infection. However, by 18 months, the exposed bone area was fully covered by epithelialized mucosa.

Panoramic radiograph at 2 years postoperatively (Figure 3C) demonstrated marked bone regeneration with restoration of mandibular continuity and resolution of the pathological fracture. At 6 years postoperatively (Figures 4A–C), no recurrence of fistula or bone exposure was observed, and the mandibular body showed sufficient bone volume and normal morphology. The patient maintained adequate oral intake without functional impairment. Although we proposed prosthetic rehabilitation, the patient declined further treatment. The underlying prostate cancer remained well controlled with continued leuprorelin therapy, and denosumab was not resumed. The patient remained under regular follow-up by the treating urologist, with no clinical or radiological evidence of new skeletal-related events (SREs) during the follow-up period.

**Figure 4:**
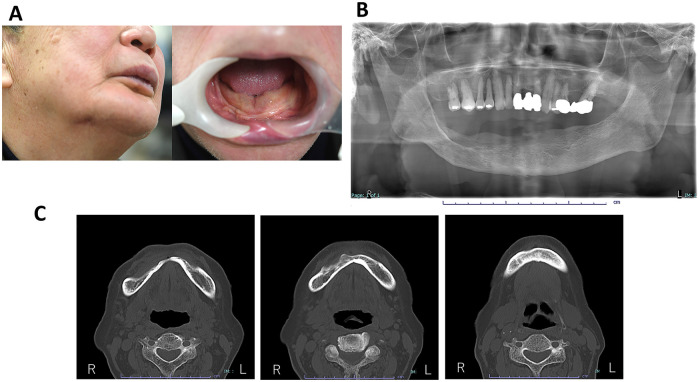
Long-term outcomes at 6 years postoperatively. **(A)** Extraoral and intraoral views showing no recurrence of fistula or bone exposure. **(B)** Panoramic radiograph showing sufficient bone volume and normal morphology of the mandibular body. **(C)** Computed tomography confirming substantial bone regeneration and structural restoration.

To provide a comprehensive overview of the clinical course, detailed timelines before and after referral to our institution are presented in Tables 1, 2, respectively.

**Table 1:** Clinical course before referral to our institution.

Date	Course at Local Dental Clinic	Course at Urology
2015	Regular dental visits of the patient are unknown.	Initial symptoms: Lower limb numbness and gait disturbance. Diagnosis: Prostate cancer with multiple bone metastases. Laboratory data: Prostate-specific antigen level was exceeding 10,000 ng/mL. Treatment: Administration of androgen deprivation therapy with a gonadotropin-releasing hormone antagonist. Posterior decompression surgery for metastatic tumors of the thoracic spine (T2–T4) was performed by the orthopedic surgery department. After transfer to rehabilitation institute, systemic therapy with enzalutamide and leuprorelin was initiated. The prostate-specific antigen level had decreased to approximately 10 ng/mL. Denosumab 120 mg was administered every 3 months.
Apr 2018	Gingival swelling in the region of tooth #46 occurred. Incision and drainage were performed. The administration of antibiotics was unknown.	—
May 2018	Occlusal pain in tooth #35 occurred. Endodontic treatment was performed.	—
Aug 2018	Numbness of the right lower lip and mental region, along with swelling of the left submandibular area developed	
Aug 2018	Gingival swelling in the region of tooth #47 occurred. Incision and drainage were performed. The administration of antibiotics was unknown.	—
Sep 2018	Gingival swelling in the region of tooth #37 and anterior mandible (#33–#43) occurred	—
Oct 2018	Gingival swelling and pus discharge in anterior mandible (#33–#43) occurred. Repeated periodontal irrigation were performed. The administration of antibiotics was unknown.	—
Sep 2019	The patent was referred to our institution due to recurrent swelling and pus discharge of the mandible.	

**Table 2:** Clinical course and management after referral to our institution.

Date	Course at Urology	Course at Our Institution		
		Clinical course and Diagnosis	Imaging Findings of the Mandible	Treatment of MRONJ
Sep 2019	Denosumab was discontinued after diagnosis of MRONJ, based on the decision of the urologist. Final administration of Denosumab was at Mar 2019. Laboratory data: the level of prostate-specific antigen was within normal range.	First visit. Diagnosis: bilateral MRONJ, stage 2, induced by denosumab. Surgical treatment planned.	Panoramic radiograph showed diffuse sclerosis and irregular trabecular patterns in the posterior mandible bilaterally, without a clear sequestrum-like appearance (Figure 1A). Computed tomography showed periosteal new bone formation along the buccal cortex and diffuse sclerosis of the cancellous bone. No definite sequestrum was identified, and mandibular continuity was preserved (Figure 1B).	Bacterial culture was performed. Amoxicillin 750 mg/day was administered for approximately 1 month. Oral hygiene instructions were provided, including regular rinsing with 0.004% benzethonium chloride between visits. Professional oral care, including mechanical cleaning and intraoral cleansing with 0.025% benzalkonium chloride solution, was performed at each visit.
Early Nov 2019	—	Rapid progression of MRONJ to stage 3 was observed several weeks before surgery. Segmental mandibulectomy was considered; however, the patient declined. Marginal mandibulectomy was selected.	Panoramic radiograph showed a sequestrum extending from the anterior mandible to the right mandibular body, reaching the inferior border (Figure 2B). Computed tomography showed widespread osteolysis and sequestrum-like changes, with findings suggestive of a pathological fracture and disruption of mandibular continuity (Figure 2C).	Levofloxacin 500 mg/day was administered for 7 days until admission.
Mid Nov 2019	—	The patient was admission to the hospital 7 days before surgery.	—	Flomoxef 2 g/day was administered intravenously until surgery. Intraoral irrigation and cleansing with antiseptic solution were performed.
Late Nov 2019	—	Marginal mandibulectomy performed under general anesthesia.	Panoramic radiograph obtained immediately after surgery showed extensive bone removal from the mental region to the right mandibular body. Only a faint outline of the remaining cortical bone was visible, and continuity of the inferior border appeared compromised (Figure 3A).	Flomoxef 2 g/day was administered intravenously until postoperative day 7.
Postoperative day 7	—	—	—	Oral amoxicillin 750 mg/day was administered for 14 days.
Postoperative day 14	—	—	—	Hyperbaric oxygen therapy was performed (total 20 sessions).
Postoperative day 38	—	The patient was discharged.	—	—
Jan 2020 (6 weeks postop)	—	Minor wound dehiscence and bone exposure were observed.	—	Intraoral irrigation and oral hygiene management, including antiseptic rinsing, were performed.
May 2020	—	The exposed bone became mobile and the pus discharge was observed.	—	Amoxicillin 750 mg/day was administered for 14 days. Oral hygiene management was continued
Oct 2020	—	Partial spontaneous sequestrum separation. Pus discharge was quitted	—	—
Jan 2021	—	Sequestrectomy was performed under local anesthesia.	—	Amoxicillin 750 mg/day was administered for 14 days.
Apr 2021 (18 months postop)	—	Complete mucosal coverage of the wound was achieved.	—	—
Nov 2021 (2 years postop)	—	Marked bone regeneration and restoration of mandibular continuity were observed.	Panoramic radiograph obtained 2 years postoperatively showed marked bone regeneration of the mandibular body, with restoration of continuity and resolution of the pathological fracture (Figure 3B).	—
Mar 2026 (6 years 5 months postop)	Denosumab was not resumed. Enzalutamide and leuprorelin were continued. Prostate -specific antigen remained within the normal range. No new metastasis was observed.	No fistula formation or bone exposure was observed.	Panoramic radiograph showed sufficient bone volume and normal morphology of the mandibular body (Figure 4B). Computed tomography showed substantial bone regeneration and structural restoration (Figure 4C).	—

## Discussion

MRONJ is a complex condition induced with the use of BMAs, and its optimal treatment remains controversial, particularly in patients with advanced malignant disease (1, 2). In such patients, treatment strategies must balance infection control, preservation of function, and the underlying oncologic condition. Surgical intervention is often required in advanced MRONJ stages; however, the extent of resection and the optimal surgical strategy remain subjects of ongoing debate, as also reflected in the position paper of the AAOMS (5, 9).

In our current patient, rapid progression of bone resorption in the mandible with MRONJ was observed after discontinuation of denosumab. Although the precise mechanism remains unclear, this phenomenon may be associated with rebound alterations in bone metabolism following cessation of antiresorptive therapy. Denosumab inhibits osteoclast activity by targeting RANKL, and its discontinuation has been reported to result in increased bone turnover and rebound skeletal events (10, 11). In contrast to high-dose bisphosphonates used in oncology, such as zoledronic acid, which accumulate in bone and continue to suppress osteoclast activity even after discontinuation, denosumab exerts its effect by reversibly inhibiting osteoclast formation through RANKL blockade (10, 11). Therefore, once denosumab is discontinued, suppressed osteoclast precursors may rapidly differentiate and become activated, potentially leading to accelerated bone resorption (10–12). Although direct evidence of such a mechanism in the present case is not available, the rapid resorption of infected and necrotic bone observed in this patient may be explained, at least in part, by reactivation of osteoclast activity following discontinuation of denosumab. Several clinical reports have also described rapid disease progression or rebound-related skeletal complications after discontinuation of denosumab (11, 12). In addition, a few reports have described worsening osteonecrosis or inflammatory bone conditions after discontinuation of antiresorptive therapy (13). These observations suggest that abrupt changes in bone remodeling dynamics may have clinically significant consequences. Although direct evidence linking this rebound phenomenon to exacerbation of MRONJ remains limited, similar mechanisms have been proposed in other bone-related conditions (10–12). Therefore, sudden restoration of osteoclast activity may contribute to accelerated bone destruction in affected regions. However, the available evidence on this topic remains limited. The observation of the present patient further supports the hypothesis that changes in bone remodeling dynamics following discontinuation of denosumab may influence the clinical behavior of MRONJ.

From a surgical perspective, marginal mandibulectomy was initially planned based on the extent of disease at the first visit. However, just prior to surgery, the area of bone resorption rapidly expanded following discontinuation of denosumab. Based on the aggravated findings, it was subsequently deemed that segmental mandibulectomy was the most appropriate treatment. However, segmental mandibular resection was not acceptable by the patient. Consequently, a limited intraoral approach was performed, in which infected- and clinically suspected necrotic bone was removed as extensively as possible while preserving the lingual bone plate and the periosteum whenever feasible. Despite the creation of a substantial structural defect, remarkable bone regeneration was observed over time. This outcome may be attributed, at least in part, to preservation of the lingual bone plate and the surrounding periosteum, which retains osteogenic potential and plays a critical role in bone healing (14). Previous experimental and clinical studies have demonstrated that the periosteum plays a key role in bone regeneration and remodeling (14, 15), although evidence in the context of advanced MRONJ remains limited. The outcome of the present patient indicates that even in extensive disease, preservation of biological structures may contribute to favorable long-term outcomes.

While these observations provide important clinical insights, several limitations should be acknowledged in the present case. First, it is not possible to attribute the rapid progression of osteolysis solely to the discontinuation of denosumab based on the data available in this study. We did not have serial measurements of bone metabolic markers, including ALP and its subtypes, NTx, ICTP, and DPD, before and after discontinuation of denosumab, during the period of rapid progression of bone resorption, or throughout the postoperative course. Second, an active infectious process, consistent with osteomyelitis superimposed on MRONJ, may also have played a substantial role in the observed clinical deterioration. In addition, although partly related to the patient's limited activities of daily living, it is difficult to conclude that sufficiently frequent and intensive oral hygiene management was achieved during the period between the initial visit and surgery. Furthermore, clinical photographs at the first visit were unavailable due to incomplete preservation of the original records, which limited retrospective assessment of the initial clinical severity and staging. Finally, the favorable outcome observed in this patient may not be solely attributable to preservation of the periosteum and residual cortical bone. Rather, it may have been influenced by multiple factors, including postoperative antibiotic therapy, hyperbaric oxygen therapy, and secondary sequestrectomy. With regard to antimicrobial management, we primarily selected penicillin-based therapy as a standard treatment for oral infections in this case. We performed antimicrobial susceptibility testing when available and confirmed the appropriateness of the selected agents. We considered this approach reasonable also from the perspective of antimicrobial resistance (AMR) stewardship. However, alternative strategies have been proposed. For example, the SIPMO-SICMF position paper suggests that combination regimens, including penicillin-based therapy with metronidazole, may be beneficial in certain cases of MRONJ, and this may warrant further consideration (16).

Another important aspect highlighted by this patient is the treatment of BMAs in patients with well-controlled metastatic malignant disease. While BMAs are effective in reducing SREs, the optimal duration of therapy and the timing of discontinuation remain uncertain (7). In clinical practice, BMAs are often continued as maintenance therapy even after disease stabilization and absence of recent SREs; however, evidence supporting indefinite continuation is limited (7, 17). In our current patient, denosumab was discontinued following the onset of MRONJ and stabilization of the underlying malignant disease, and it was not resumed thereafter. Notably, no recurrence of MRONJ or new SREs was observed during long-term follow-up. This clinical course may suggest that continued BMA therapy may not always be necessary once metastatic disease is controlled and SREs are no longer evident. Although BMAs play a critical role in the prevention of SREs (7), prolonged use is associated with several cumulative risks, including MRONJ (1, 2). Therefore, individualized treatment strategies—taking into account disease activity, prior SREs, and the risk of adverse events—may be required. However, it should be noted that the patient's preference played a significant role in the treatment decision in this case. At the same time, we recognize that indiscriminate downgrading of surgical treatment in stage 3 MRONJ may be inappropriate. The choice and extent of surgical intervention should be determined carefully, taking into account the disease status of MRONJ as well as the overall condition of the patient.

In addition, this case highlights important challenges in the prevention and early recognition of MRONJ. As reflected in the clinical course, appropriate preventive dental care and early identification of MRONJ-related symptoms were not fully achieved. This may be related, at least in part, to insufficient awareness of BMAs and MRONJ, as well as limited communication between dental and medical practitioners. Such issues are not unique to this case but represent broader challenges in the management of patients receiving BMAs. Therefore, close interdisciplinary collaboration between oncologists and oral and maxillofacial surgeons, along with appropriate patient education and preventive dental care, is essential to optimize treatment decisions and reduce the risk of MRONJ in such complex cases.

## Conclusions

Rapid progression of bone resorption was observed in a patient with MRONJ, in which discontinuation of denosumab may have been one of the contributing factors. In addition to multidisciplinary management, including oral hygiene care and pharmacological therapy, preservation of biological structures such as the lingual bone plate and the periosteum during marginal mandibulectomy may be associated with favorable bone regeneration even in advanced MRONJ. These findings highlight the complexity of MRONJ management and the importance of individualized, multidisciplinary approaches in patients with metastatic malignant disease.

## Data Availability

The raw data supporting the conclusions of this article will be made available by the authors, without undue reservation.
